# Robotic spinal angiography: A single-center experience

**DOI:** 10.1177/15910199241272515

**Published:** 2024-08-08

**Authors:** Oleg Shekhtman, Georgios S Sioutas, Mert Marcel Dagli, Irina-Mihaela Matache, Bryan A Pukenas, Brian T Jankowitz, Jan-Karl Burkhardt, Visish M Srinivasan

**Affiliations:** 1 189491 Department of Neurosurgery, 14640 Perelman School of Medicine, 6572 University of Pennsylvania, Philadelphia, Pennsylvania, USA; 2 Department of Physiology, 87267 Carol Davila University of Medicine and Pharmacy, Bucharest, Romania; 3 Department of Radiology, 14640 Perelman School of Medicine, 6572 University of Pennsylvania, Philadelphia, Pennsylvania, USA; 4 24263 Hackensack Meridian Neuroscience Institute at JFK University Medical Center, Edison, New Jersey, USA

**Keywords:** Robotic-assisted procedures, spinal angiography, dural arteriovenous fistula

## Abstract

**Background and objectives:**

Robotic neurointervention enhances procedural precision, reduces radiation risk, and improves care access. Originally for interventional cardiology, the CorPath GRX platform has been used in neurointerventions. Recent studies highlight robotic cerebral angiography benefits, but information on spinal angiography is limited. While a new generation of robotic solutions is on the horizon, this series evaluates our experience with the CorPath GRX in spinal angiographic procedures, addressing a key gap in neurointerventional research.

**Methods:**

In this single-center retrospective case series, we analyzed 11 patients who underwent robotic-assisted diagnostic procedures with the CorPath GRX system from February 2022 to March 2023 at our institution. A descriptive synthesis was performed on the demographic, baseline, surgical, and postoperative data collected.

**Results:**

The average age of the 11 patients was 54 ± 20.34 years, with six (54.55%) female. The mean body mass index was 29.58 ± 7.86, and 7 (63.64%) were non-smokers. Of the 11 procedures using the CorPath GRX system, four (36.36%) were partially converted to manual technique. General anesthesia was used in nine cases (81.82%), and right-side femoral access in ten (90.91%) patients. Mean fluoroscopy time was 24.81 ± 10.19 min, contrast dose 174.09 ± 57.31 mL, dose area product 472.23 ± 437.57 Gy·cm², and air kerma 2438.84 ± 2107.06 mGy. No robot-related complications and minimal procedure-related complications were reported.

**Conclusion:**

The CorPath GRX system, a robotic-assisted platform, has proven reliable and safe in spinal angiography, evidenced by its enhanced procedural accuracy and reduced radiation exposure for operators.

## Introduction

The integration of robotic assistance into neuro-endovascular procedures marks a potentially transformative era in patient care. The CorPath GRX robotic-assisted platform (Corindus Inc., Waltham, USA) is a potential frontrunner in this paradigm shift. Originally designed for interventional cardiology, the system's robustness has been validated through rigorous clinical trials, leading to its approval by the U.S. Food and Drug Administration (FDA) and the acquisition of the Conformité Européenne (European Conformity) mark for performing percutaneous coronary interventions. While the current version is being discontinued, new robotic solutions may be expected in the near future. The advent of such technology heralds a new chapter not only in cardiology but also in the broader realm of interventional medicine, suggesting a potential crossover into other complex areas like neurointervention.

Robotic neurointervention is an evolving technology that has the potential to improve procedural precision, reduce occupational radiation risk, and increase access to care. Although robotic endovascular technology is still in its early stages, teleoperation features of such systems could surmount geographical barriers, offering a lifeline to remote and underserved populations in need of timely neurovascular care. While the academic discourse has begun to spotlight the benefits of robotic assistance in cerebral angiography, the specific application to spinal angiography remains notably underreported. This paucity of data underscores an urgent need for empirical research to assess the feasibility and outcomes of such interventions.

This study aims to address this gap by analyzing the application of the CorPath GRX platform in spinal angiography. Through a case series at a single academic institution, our investigation seeks to provide preliminary insights into the safety and efficacy of robotic technology in spinal angiographic procedures.

## Methods

### Study design

We conducted a single-center retrospective case series of patients undergoing spinal angiography with the CorPath GRX Robotic System. Patient data were extracted from the electronic health record system. Patients were admitted for elective spinal diagnostic angiography or interventional procedures from February 2022 to March 2023 at our center. The study was approved by the Institutional Review Board, and individual consent was waived due to the retrospective design of the study.

### Procedure description

The patients were positioned supine on the angiogram table, prepared, and draped in the standard sterile manner. Diagnostic spinal angiogram procedures were conducted under general anesthesia or monitored anesthesia care (MAC). Standard right femoral access was obtained with a 5F sheath.

The selection catheters (5F Mikaelsson or 5F HS-1) were inserted manually into the sheath over a wire and formatted. The catheters were then withdrawn to the mid-point spinal level of the desired region of investigation to allow 10 cm of catheter translation in each direction rostral–caudal. Subsequently, the catheter was loaded on the robotic cassette. A dedicated robotic technologist and an assistant neurointerventionalist handled the loading and exchange of devices within the robotic system, while the primary neurointerventionalist operated at the workstation. Maneuvers included rostral–caudal translation and rotation. The catheter was also connected to a power injector such that the interventionalist could do “puffs” of contrast while searching for the ostium of arteries. The power injection protocol for each run was 1 cm^3^/s for 2 s.

### Device description

The CorPath GRX Robotic System (Corindus, a Siemens Healthineers Company, Waltham, Massachusetts, USA) consists of two main components: a remote physician unit and a bedside unit. The bedside unit includes an articulated arm, a robotic drive, and a single-use disposable cassette. The cassette serves as the mechanical transmission module, translating real-time commands from the remote physician unit's designated joysticks to manipulate the devices. This functionality allows the operator to advance, retract, and rotate catheters and wires. The robotic system is compatible with 0.014 and 0.018-in. guidewires and exchange catheters, balloons, and stent catheters (Figure 1).

**Figure 1.**
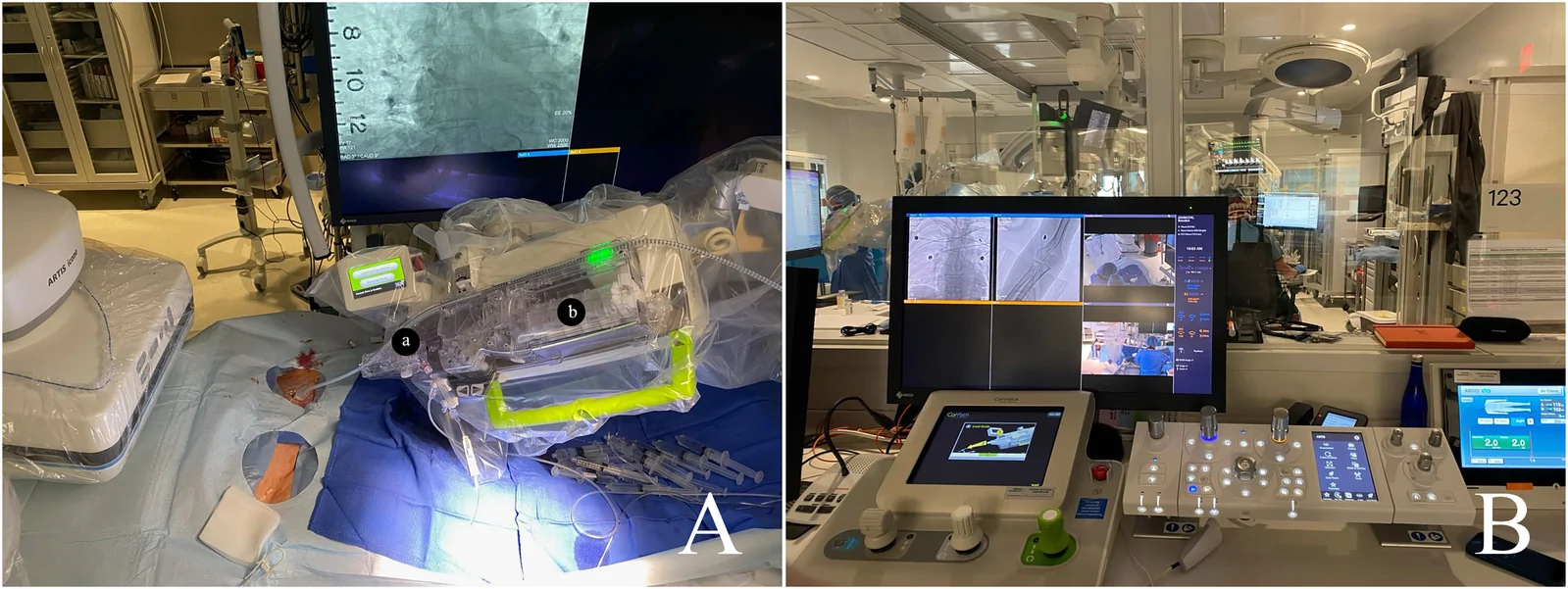
(A) The CorPath GRX Robotic System: a = Y-connector, b = cassette with linear and rotational drives; and (B) operation platform.

The robotic console is situated outside the control room with direct visualization of the table and angiography machine. It features a 26-in. monitor for viewing biplane live fluoroscopic views, along with three joysticks and touchscreen controls. The robotic system is equipped with the capability to independently advance, retract, and rotate the catheter and guidewire. Additionally, it has the capacity to deploy either an additional rapid-exchange device or a guidewire.

### Data collection and statistical analysis

Demographic and baseline characteristics were collected, along with surgical/radiographic data (anesthesia type, access, access catheter, access wire, conversion to manual angiography, procedure time, fluoroscopy time, contrast dose, dose area product (DAP), air kerma, and selected vessels. Outcome measures included robot-related complications, procedure-related complications, and in-hospital complications were extracted from patient records. A descriptive synthesis of the observations was performed. Continuous variables were summarized with mean and standard deviations. Categorical parameters were described by frequency counts and percentages.

## Results

### Patient characteristics

A total of 11 patients underwent spinal angiography with the CorPath GRX Robotic System and were included in the study. The mean age was 54 ± 20.34 years. Six (54.55%) patients were female and the majority were white (81.82%). The mean body mass index (BMI) was 29.58 ± 7.86 and seven (63.64%) patients were non-smokers. At admission, diagnoses were: three (27.27%) dural arteriovenous fistulas (dAVFs), two (18.18%) vascular malformations, two (18.18%) epidural hematomas, and four (36.36%) spinal lesions. Patient demographics and baseline characteristics are presented in Table 1.

**Table 1.** Patient characteristics.

Variable	Patients (*n* = 11)
Age (years)	54 ± 20.34
Sex	
Female	6 (54.55%)
Male	5 (45.45%)
Race	
White	9 (81.82%)
African American	1 (9.09%)
Unknown	1 (9.09%)
BMI	29.58 ± 7.86
Smoking status	
Non-smoker	7 (63.64%)
Ex-smoker	4 (36.36%)
Diagnosis at admission	
dAVF	3 (27.3%)
Vascular malformation	2 (18.2%)
Epidural hematoma	2 (18.2%)
Spinal lesion	4 (36.4%)

BMI: body mass index; dAVF: dural arteriovenous fistula.

### Robotic-assisted spinal angiography

A total of 11 procedures were performed. The mean procedure time was 70.36 ± 17.7 min; the majority of patients received general anesthesia (81.82%) and femoral access through the right side (90.91%). The mean fluoroscopy time was 24.81 ± 10.19 min, the mean contrast dose was 174.09 ± 57.31 mL, the mean DAP was 472.23 ± 437.57 Gy·cm², and the mean air kerma was 2438.84 ± 2107.06 mGy. The most common access catheter used was the 5-French HS-1 catheter (54.5%), followed by the 5-French Mikelson catheter (36.4%). Surgical details are presented in Table 2.

**Table 2.** Surgical details.

Variable	Patients (*n* = 11)
Procedure duration (min)	70.36 ± 17.7
Anesthesia type	
General	9 (81.82%)
MAC	2 (18.18%)
Femoral access	
Right	10 (90.91%)
Left	1 (9.09%)
Access catheter	
5-French HS-1	6 (54.5%)
5-French Mikelson	4 (36.4%)
5-French angled catheter	1 (0.9%)
Fluoroscopy time (min)	24.81 ± 10.19
Contrast dose (mL)	174.09 ± 57.31
Dose area product (Gy·cm²)	473.23 ± 437.57
Average air kerma (mGy)	2438.84 ± 2107.06
Mean no. vessels contrasted	22.27 ± 8.86
Conversion to manual procedure	4 (36.4%)

MAC: monitored anesthesia care.

### Clinical case

An adult male presented to the emergency room with an acute onset of lower extremity paralysis in the setting of 2–3 weeks of low paresthesia. Magnetic resonance imaging revealed a T12-L2 vascular lesion. During the spinal robotic-assisted angiography, a spinal arteriovenous malformation (AVM; Type 4b) was identified, with a nidus centered at the thoracolumbar junction measuring approximately 3.6 × 0.7 cm. AVM was primarily supplied by hypertrophied branches of the anterior spinal artery arising from the left T9 segmental artery and prominent branches of the posterior spinal artery arising from the right T12 segmental artery. Minor contribution is also seen from radiculopial/radiculomedullary arterial branches arising from the left L2 segmental artery. The AVM was treated with a combination of Onyx embolization and dural vein clipping (Figure 2).

**Figure 2.**
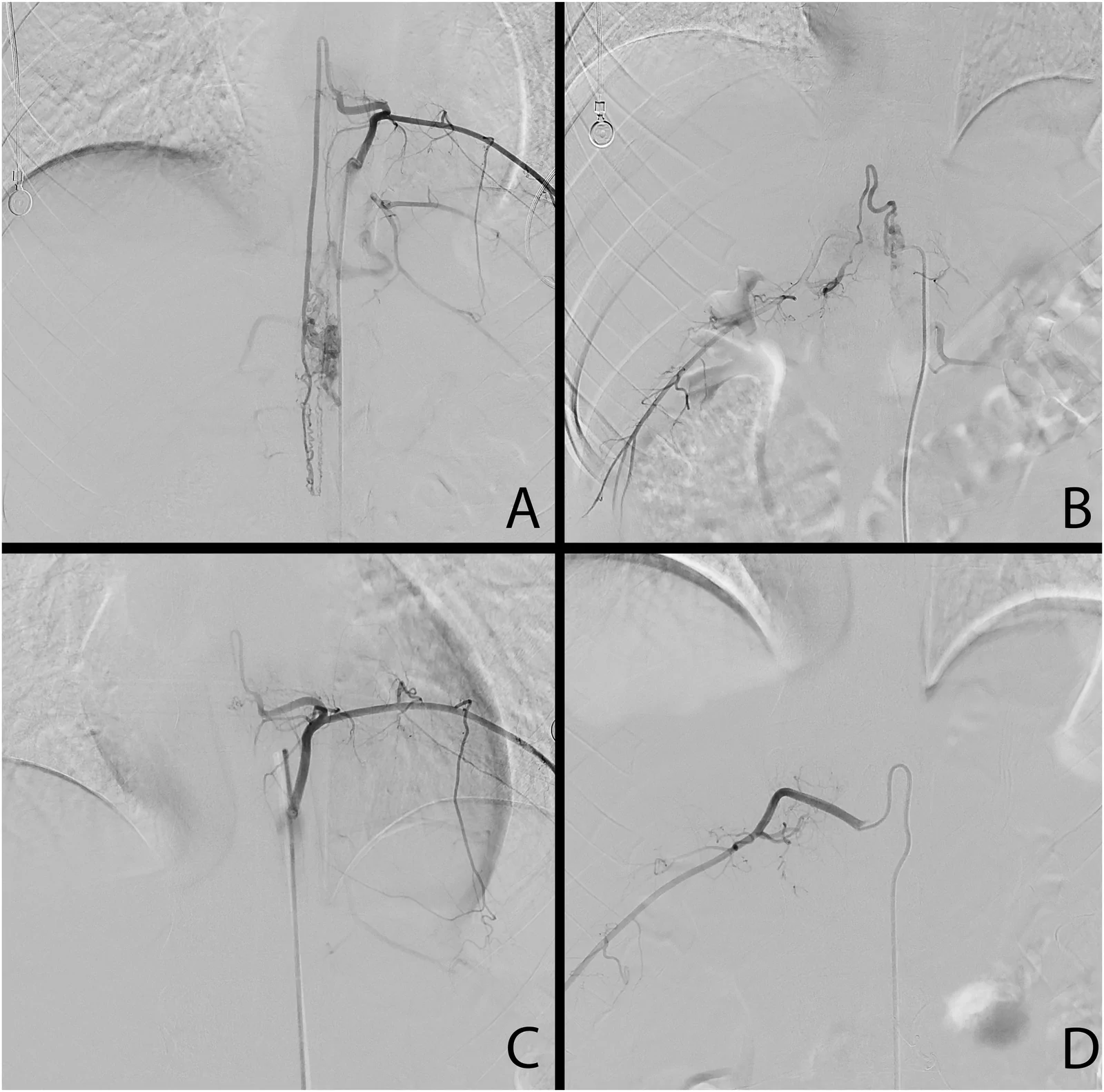
Type IVb arteriovenous malformation (AVM) was primarily supplied by hypertrophied branches of the anterior spinal artery arising from the left T9 segmental artery and prominent branches of the posterior spinal artery arising from the right T12 segmental artery (A, B). AVM was treated with a combination of Onyx embolization and dural vein clipping (C, D).

### Outcomes and complications

All diagnostic procedures were completed. There were no robot-related, procedure-related, or in-hospital complications. In four patients the procedure was converted to manual due to anatomic complexity or incomplete catheterization with robotic assistance.

## Discussion

In the present study, we analyzed 11 cases of our experience utilizing the latest CorPath GRX robotic-assisted platform to perform spinal angiography with the main purpose of understanding the major benefits of this promising technology. To our knowledge, there is only one case report on the same topic. Tateshima et al. employed robotic angiography to identify a T4 vertebral body metastatic tumor supplied by bilateral T3–5 segmental arteries.^1^

The radiological parameters observed in our series were consistent with those reported in the literature. The mean DAP in our series was 477.23 Gy·cm^2^, the mean fluoroscopy time 24.81 min, and the mean contrast volume 174.09 mL. In a study by Ozpeynirci et al.,^2^ the mean procedural DAP for 62 spinal angiographies performed in 25 patients with spinal dAVFs was 260 Gy·cm^2^, with an average fluoroscopy time of 19.6 min and a mean volume of contrast material used of 143 mL. Radiation doses seen in endovascular procedures are typically higher than those from diagnostic angiographies due to the increased number of injections involved. Opitz et al. studied diagnostic reference levels in a large dAVF series, including 37 patients with spinal dAVF.^3^ Authors reported mean DAP and fluoroscopy time for diagnostic angiography as 299.5 Gy·cm^2^ and 25 min versus 347.12 Gy·cm^2^ and 35 min for neurointerventional procedures, respectively. Radiation exposure could be significantly decreased if dAVF levels are already established. Luetmer et al. demonstrated that fluoroscopy time and the volume of contrast agent were reduced by more than 50% in the 13 patients with a spinal dAVF in whom magnetic resonance angiography prospectively indicated the correct level.^4^

We have not encountered any periprocedural complications, likely due to the limited case volume. A review of the literature indicates that complication rates for spinal digital subtraction angiography (DSA) fall within the range of 0.7 to 2.2%. These complications encompass general issues including nausea, vomiting, contrast allergy, stroke, and groin hematoma, as well as specific complications including mono- and paraparesis, back spasms, and decreased sensation.^5,6^

In four cases, robotic-assisted procedures were converted to manual. Published data on this matter is very scarce. Beaman et al. documented a 22.1% conversion rate in 113 cerebral angiography cases.^7^ Principal causes for unplanned manual conversion included challenging anatomy, limited working length, cassette or robotic arm failures, or console errors. Our experience with Corindus is rather positive; once the learning curve is passed, procedure time and convenience improve. All four of the manual conversions in our series were attributed to the desire for additional vessel catheterization. The travel distance of 20 cm total on the robotic cassette precludes a complete spinal angiogram. In the seven procedures that did not require manual conversion, critical vessels (anterior spinal, posterior spinal) and lesions were identified within the 20 cm zone. One may expect the technical durability of Corindus to improve with the new generation system. As a reference, Koh et al. reported mechanical failures in 10,000 robotic da Vinci procedures equal to only 1.8%.^8^

Corindus Robotic System was FDA-cleared in the USA for percutaneous coronary in 2012 and peripheral vascular interventions in 2018.^9^ The safety and feasibility of robotic-assisted platforms for percutaneous coronary intervention and later for peripheral artery disease were demonstrated in pivotal trials.^10,11^ The feasibility of using the Corpath GRX for neurovascular interventions has been first demonstrated in preclinical studies. Britz et al. successfully navigated and deployed small-gauge devices (stent and coils) without encountering any technical complications in a swine model.^9^ Desai et al. were the first to demonstrate the use of Corindus for intracranial AVM embolization in a swine model.^12^ Furthermore, few reports have described 100% CorPath GRX robotic-assisted procedures for transradial carotid angiography, carotid angioplasty, and carotid stenting, including the application of a distal embolic protection device.^13–15^

The exploration of neurovascular applications is actively progressing, supported by a recent prospective multicenter clinical trial that showcased the effectiveness and safety of the CorPath GRX System in endovascular embolization procedures for cerebral aneurysms. Out of the 117 patients enrolled, primary effectiveness (successful completion of the procedure) was achieved in 110 out of 117 (94%) subjects, with only seven patients requiring conversion to manual surgery, two intraprocedural aneurysm ruptures, and two strokes.^16^ Chivot et al. recently published a series involving 10 patients undergoing robot-assisted flow diverter stent deployment with the CorPath GRX platform.^17^ All flow diverter stents were successfully deployed, with only one instance of partial conversion to a manual technique due to guidewire torquability limitations. Cancelliere et al. achieved complete occlusion or the desired coil packing in six cases of aneurysms, applying complex maneuvers such as changing the microcatheter position inside the sac or re-entering the aneurysm after being pushed out.^18^

The absence of haptic feedback in current endovascular robots is a notable limitation, although there are indications that this could be addressed in the next generation of robots. A significant drawback of all existing robotic platforms is the need for manual assistance at various stages of the procedure. The current versions of the Corpath GRX do not support the robotic deployment of devices or the robotic inflation of balloons for angioplasty.^19^ The majority of robotically performed stages are confined to navigating catheters, wires, or angioplasty balloons to the target site and deploying and removing distal embolic protection devices.^19^ Another hindrance to the widespread adoption of robotic surgery is the costs, which may be difficult to justify in certain socioeconomic/payor contexts.^20^

Several considerations should be taken into account when starting a CorPath GRX-assisted procedure. Firstly, atherosclerotic plaques or anatomical variations may increase the complexity of the procedure when using a robotic approach. Secondly, all instruments need to be loaded manually by the assisting personnel, thus making the procedure somewhat semi-automated.

Nevertheless, a key advantage of robotic-assisted spinal DSA comes from anatomy. The origins of the segmental arteries position in line, making catheter navigation noticeably easier. Our experience shows that robotic navigation allows for precise movement of the catheter tip along the desired axis, eliminating rotational deviations that often occur with manual guidance. It is straightforward to return to a prior catheter position as the robot tracks the degree of rotation and distance of catheter travel. Thus, if one gets off-axis with the origin of the segmental arteries, it is possible to refer back to the location (e.g. at L1, it was −10° on the left and +10° on the right). Next, positioning the interventionalist in a seated position gives ergonomic advantages that one sees in microsurgery (cited in my microsurgery survey paper). Further, with the large screen directly in front of the neurointerventionalists, there are subjective improvements in visualization and radiologic focus. Lastly, because the movements are far more precise, we find that one has “less of a lead thumb” (less contrast use).

### Future perspectives

The accumulation of clinical experience and the emergence of next-generation robotic platforms with improved features may serve as a foundation for enhancing practices. A promising solution could involve further development of automated maneuvers, in addition to the existing rotate on retract (RoR) and “active device fixation”.^20^ These automated maneuvers could be optimized based on spinal computed tomography angiography (CTA) imaging before DSA. Hashikata et al. utilized spinal CTA images to identify bifurcations of the segmental arteries and then merged the data with a multiwindow DSA display to guide the catheter for embolizing thoracolumbar fistula.^21^ The diagnostic accuracy of the spinal DSA may be enhanced by fusing it with three-dimensional magnetic resonance images.^22^

### Study limitations

The study carries certain limitations primarily due to its small size and retrospective nature. The limited sample size of this single-center study may not adequately represent the diversity present in larger populations. Because patients were not randomly chosen, this study might have selection bias. Additionally, we did not directly compare robotic to manual operations. Lastly, it should be noted that the CorPath GRX robotic system is not FDA-approved for spinal angiography specifically and that this use was off-label as an extrapolation of diagnostic cervicocerebral angiography.
